# Tailoring a Plasmodium vivax Vaccine To Enhance Efficacy through a Combination of a CSP Virus-Like Particle and TRAP Viral Vectors

**DOI:** 10.1128/IAI.00114-18

**Published:** 2018-08-22

**Authors:** Erwan Atcheson, Karolis Bauza, Ahmed M. Salman, Eduardo Alves, Joshua Blight, Martha Eva Viveros-Sandoval, Chris J. Janse, Shahid M. Khan, Adrian V. S. Hill, Arturo Reyes-Sandoval

**Affiliations:** a The Jenner Institute, University of Oxford, Oxford, United Kingdom; b Leiden Malaria Research Group, Department of Parasitology, Center of Infectious Diseases, Leiden University Medical Center, Leiden, The Netherlands; c Laboratorio de Hemostasia y Biología Vascular, División de Estudios de Posgrado, Facultad de Ciencias Médicas y Biológicas Dr. Ignacio Chávez, Universidad Michoacana de San Nicolás de Hidalgo (UMSNH), Morelia, Michoacán, Mexico; d UMSNH-Oxford University Clinical Research Laboratory, Faculty of Biological and Medical Sciences Dr. Ignacio Chávez, Universidad Michoacana de San Nicolás de Hidalgo, Morelia, Michoacán, Mexico; University of South Florida

**Keywords:** Plasmodium vivax, VLP, adenoviruses, malaria, vaccines

## Abstract

Vivax malaria remains one of the most serious and neglected tropical diseases, with 132 to 391 million clinical cases per year and 2.5 billion people at risk of infection. A vaccine against Plasmodium vivax could have more impact than any other intervention, and the use of a vaccine targeting multiple antigens may result in higher efficacy against sporozoite infection than targeting a single antigen. Here, two leading P. vivax preerythrocytic vaccine candidate antigens, the P. vivax circumsporozoite protein (PvCSP) and the thrombospondin-related adhesion protein (PvTRAP) were delivered as a combined vaccine. This strategy provided a dose-sparing effect, with 100% sterile protection in mice using doses that individually conferred low or no protection, as with the unadjuvanted antigens PvTRAP (0%) and PvCSP (50%), and reached protection similar to that of adjuvanted components. Efficacy against malaria infection was assessed using a new mouse challenge model consisting of a double-transgenic Plasmodium berghei parasite simultaneously expressing PvCSP and PvTRAP used in mice immunized with the virus-like particle (VLP) Rv21 previously reported to induce high efficacy in mice using Matrix-M adjuvant, while PvTRAP was concomitantly administered in chimpanzee adenovirus and modified vaccinia virus Ankara (MVA) vectors (viral-vectored TRAP, or vvTRAP) to support effective induction of T cells. We examined immunity elicited by these vaccines in the context of two adjuvants approved for human use (AddaVax and Matrix-M). Matrix-M supported the highest anti-PvCSP antibody titers when combined with Rv21, and, interestingly, mixing PvCSP Rv21 and PvTRAP viral vectors enhanced immunity to malaria over levels provided by single vaccines.

## Abstract (precis)

Vivax malaria remains one of the most serious and neglected tropical diseases, with 132 to 391 million clinical cases per year and 2.5 billion people at risk of infection. A vaccine against Plasmodium vivax could have more impact than any other intervention, and the use of a vaccine targeting multiple antigens may result in higher efficacy against sporozoite infection than targeting a single antigen.

## INTRODUCTION

Vivax malaria remains one of the world's most neglected tropical diseases (1, 2), and it is considered to be responsible for 132 to 391 million clinical cases per year with 2.5 billion people at risk of infection (3), mainly in Southeast Asia and Latin America (4). An efficacious vaccine against Plasmodium vivax or Plasmodium falciparum could have more impact in reducing or eliminating this disease than any other intervention (5, 6). However, parasite diversity and a complex life cycle are major obstacles hampering vaccine development, and it is considered that an efficacious vaccine may require harnessing cellular and humoral immune responses against multiple antigens (7). This view has prompted an evaluation of the efficacy of multiple antigens using various vaccine platforms against infection by Plasmodium berghei (8) and P. falciparum (9).

The leading vaccine candidates against P. vivax are the preerythrocytic antigen P. vivax circumsporozoite protein (PvCSP) (2, 10, 11) and the blood stage antigen Duffy binding protein (12, 13), both having completed clinical trials (https://clinicaltrials.gov/show/NCT01816113) (10). The low levels of protective efficacy of the PvCSP vaccine candidate VMP001 has prompted the development of improved strategies consisting of a particulate circumsporozoite antigen delivered as a virus-like particle (VLP), CSV-S,S (14), and Rv21 (15). The thrombospondin-related adhesion protein (TRAP) is another preerythrocytic vivax malaria antigen that has shown promise in a recent preclinical study (16). Effective preerythrocytic vivax vaccines, unlike blood stage approaches, would have the potential to prevent or eliminate the quiescent liver stage forms known as hypnozoites, which cause relapse long after a primary infection (17) and are among the greatest obstacles to malaria eradication (2).

For P. falciparum malaria, similar vaccine candidates based on CSP (PfCSP) and PfTRAP have a longer track record of development than their P. vivax counterparts, but, unfortunately, both have reported suboptimal protection in humans (18). The leading RTS,S/AS01 falciparum vaccine candidate, Mosquirix, has completed phase III clinical trials, but despite its approval by the European Medicines Agency for childhood immunization, it did not achieve an endorsement by the WHO, thus limiting its future integration into existing malaria control programs (19). The most recent results indicate that three vaccine doses of RTS,S/AS01 protected 37% of infants (20) and 47% of children (21) against severe malaria. In a recent phase IIa clinical trial, PfTRAP delivered in a chimpanzee adenovirus 63 and modified virus Ankara (ChAd63-MVA) prime-boost regime induced sterile protection in 21% of human volunteers (22).

Efforts to improve the efficacy of malaria vaccines have led to the development of vaccine approaches in which not a single antigen is targeted; instead multiple antigens, either from the same life cycle stage or from multiple stages, are targeted. In this regard, vaccine approaches targeting the two leading preerythrocytic vaccine candidates, CSP and TRAP, show enhanced protection in animal models of malaria (8, 23) compared to vaccines targeting a single antigen. A recent phase IIa clinical trial evidenced an enhancement in protective efficacy against challenge with P. falciparum using a combination of RTS,S/AS01B with recombinant viral vectors ChAd63 and MVA expressing ME-TRAP (where ME is multiepitope) (9), thus supporting this combination strategy.

A model of enhanced protection using a vaccine targeting CSP and TRAP was suggested by Bauza et al. (8), using a two-stage approach, whereby antibodies (Abs) to P. berghei CSP (PbCSP) limited the number of P. berghei sporozoites reaching the liver, followed by PbTRAP-specific CD8^+^ T cells destroying a reduced number of infected hepatocytes. Viral vectors encoding the target antigen have been shown to elicit high numbers of CD8^+^ T cells, particularly when adenovirus-modified vaccinia virus Ankara (Ad-M or viral-vectored TRAP [vvTRAP]) is used in heterologous prime-boost regimens (16, 22, 24). Thus, viral vectors are used here to deliver the PvTRAP antigen (vvTRAP) to stimulate cytotoxic T-lymphocyte (CTL) responses, whereas the VLP Rv21 was used to generate high titers of anti-CSP antibodies (Abs). Rv21 VLP is based on the hepatitis B surface antigen (HepBsAg), and it has been shown to be highly efficacious against a sporozoite challenge, providing complete, sterile protection following two doses of Rv21 in Matrix-M adjuvant (15).

While vaccination using two antigens would be expected to provide enhanced protection against infection, the possibility remains of inducing antigenic interference due to the use of adjuvants or platforms to deliver the antigens. The phenomenon of antigenic interference may hamper the success of any combination vaccine due to the decrease in antibody or cytotoxic T-cell responses against any of the vaccine antigens, which has been observed in studies where two or more antigens have been combined (8, 25). Here, we aimed to augment the efficacy of known vivax preerythrocytic vaccines by combining CSP and TRAP using two different vaccine platforms for each antigen and two adjuvants suitable for human use: (i) AddaVax, a squalene-based oil-in-water nanoemulsion with a formulation similar to that of MF59 (26), and (ii) Matrix-M, a nanoparticle saponin-based adjuvant (27, 28).

## RESULTS

In order to assess *in vivo* protective efficacy of the different P. vivax vaccine platforms, we developed a rodent challenge model consisting of a double-chimeric P. berghei parasite line that was generated by replacing the *Pbtrap* with the *Pvtrap* gene in a single chimeric line in which the *Pbcsp* gene had initially been replaced by the VK210 allele of *Pvcsp*. The single chimeric line, PbANKA-PvCSP-VK210(r)_PbCSP_ (line 2196cl1), was developed through the use of gene insertion/marker out (GIMO) transfection technology as described previously (15) and is free of a drug-selectable marker. In addition, the resulting parasite contains the green fluorescent protein (GFP)-luciferase fusion gene as a reporter. The absence of a drug-selectable marker permitted the replacement of the *Pbtrap* gene with the *Pvtrap* gene in parasites of the PbANKA-PvCSP-VK210(r)_PbCSP_ line in a single transfection experiment using a *trap* replacement construct described previously (16). This construct contains a synthetic *trap* allele composed of the protein-coding sequence of P. vivax TRAP (NCBI accession number XM_001614097) from the Salvador I strain that was codon optimized for expression in P. berghei using GeneOptimizer software. Transfection of PbANKA-PvCSP-VK210(r)_PbCSP_ parasites with the *trap* replacement construct and positive selection with pyrimethamine resulted in selection of double-chimeric parasites [PbANKA-PvCSP-VK210(r)_PbCSP_-PvTRAP(r)_PbTRAP_], referred to as PvCSP-VK210/PvTRAP parasites (see Fig. S1 in the supplemental material). Integration of the *Pvcsp* and *Pvtrap* genes in PvCSP/PvTRAP was confirmed by diagnostic Southern analysis of chromosomes separated by pulsed-field gel electrophoresis and diagnostic PCR on genomic DNA (gDNA) (Fig. S2A and B). PvCSP-VK210/PvTRAP parasites are double-chimeric P. berghei parasites that do not contain the P. berghei *csp* or *trap* gene coding sequence (CDS) but express both P. vivax *csp-VK210* and the P. vivax *trap* CDS under the control of the *P. berghei csp* and *trap* equivalent regulatory sequences and is drug-selectable marker free.

**FIG 1:**
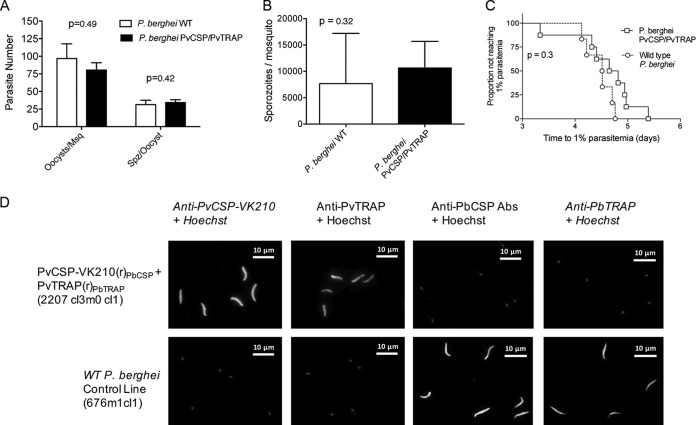
Parasite fitness and phenotype characterization of a double-transgenic P. berghei parasite expressing Plasmodium vivax CSP-VK210 and TRAP. The ability of the PvCSP-VK210/PvTRAP double-transgenic parasite to develop in the invertebrate host was evaluated. (A) Ten days after feeding on infected mice, mosquito midguts were obtained, and oocysts were enumerated for both wild-type and PvCSP-VK210/PvTRAP 2207 cl3m0cl1 parasites. (B) Twenty-one days after feeding, salivary gland sporozoites were extracted and enumerated, and the numbers of sporozoites per mosquito was compared between wild-type and PvCSP-VK210/PvTRAP 2207 cl3m0cl1-infected Anopheles stephensi. Data are shown as means with standard deviations, and *P* values from *t* tests. (C) The ability of 2207 cl3m0cl1 to infect vertebrate hosts was evaluated by injecting salivary gland sporozoites into naive BALB/c mice (*n* = 6) and monitoring for blood stage infection. Outcome is shown as the time to reach 1% blood stage parasitemia. A Mantel-Cox test was performed between groups. (D) Transgenic salivary gland sporozoites were stained with anti-PvCSP-VK210, polyclonal anti-PvTRAP, or with serum from vaccinated mice with ChAd63-MVA expressing PvTRAP or PbTRAP. Alexa Fluor 488-labeled anti-mouse IgG and Hoechst-33342 (nuclear staining) were also included for the assay. As a control, wild-type (WT) P. berghei sporozoites were stained with the same antibodies. Merged images of the different channels are shown for both transgenic and WT P. berghei stained images.

We next assessed the ability of the double-replacement chimeric parasites to produce oocysts and sporozoites since replacing the endogenous *csp* and *trap* genes with the P. vivax orthologs could alter these characteristics. Oocyst and sporozoite production in Anopheles stephensi of PvCSP-VK210/PvTRAP was similar to oocyst and sporozoite production of wild-type (WT) P. berghei (Fig. 1A and B). The infectivity of sporozoites of both transgenic parasites was assessed by determination of the prepatent period (i.e., the time to 1% parasitemia) after intravenous injection of 2,000 sporozoites in the tail vein of BALB/c mice. All mice developed blood parasitemia, and the times to reach 1% parasitemia were not different between WT and PvCSP-VK210/PvTRAP parasites (Fig. 1C). Expression of the *Pvcsp-VK210* and *Pvtrap* proteins in PvCSP-VKCSP/PvTRAP sporozoites was confirmed by immunofluorescence microscopy of sporozoites using anti-PvCSP monoclonal antibodies to the repeat region of the PvCSP-VK210 and polyclonal anti-PvTRAP antibodies. PbTRAP was present only in wild-type sporozoites and not in PvCSP-VK210/PvTRAP sporozoites (Fig. 1D). These results demonstrate that the double-chimeric PvCSP-VK210/PvTRAP parasites produce infectious sporozoites, which are able to complete full liver stage development in mice.

Next, we used these chimeric PvCSP-VK210/PvTRAP sporozoites as a challenge model by infecting immunized mice and assessing protective efficacy of various combinations of PvCSP-VK210 and PvTRAP vaccines.

### Combining vvTRAP with vivax CSP enhances protection compared to that of either component alone.

**FIG 2:**
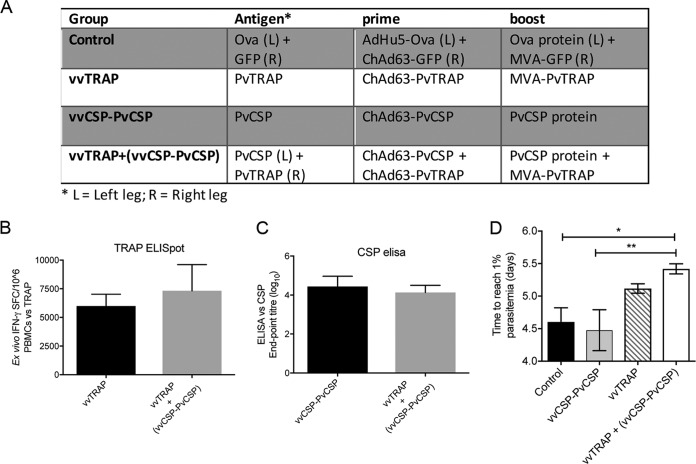
Immunogenicity and protective efficacy of a combination P. vivax CSP-VK210 with adenoviral or MVA viral vectors expressing P. vivax TRAP (vvTRAP). (A) Inbred female BALB/c mice (*n* = 7 to 8/group) were primed with PvCSP-VK210 viral vector (vv) followed by the PvCSP protein (vvCSP-PvCSP), ChAd63-MVA viral vector expressing PvTRAP (vvTRAP), or their combination (vvTRAP+vvCSP-PvCSP). (B) Two weeks postboost *ex vivo* IFN-γ ELISpot responses were measured upon restimulation of whole-blood PBMCs with three subpools of peptides representing the full-length PvTRAP. Values are means and standard errors of the means. (C) An endpoint titer ELISA was done to quantify anti-PvCSP-VK210 antibodies. Bars indicate median responses. (D) Two weeks after the last vaccination, animals were challenged by intravenous injection of 2,000 double-transgenic P. berghei PvCSP-VK210 and PvTRAP sporozoites. Blood stage infection was recorded over three consecutive days starting at day 5 postchallenge, and a linear regression model predicting time to 1% blood stage parasitemia for individual animals was applied, showing a significant delay in the vvTRAP+vvCSP-PvCSP group that received a combination vaccine compared to time for the other treatment groups. Values are means and standard errors of the means. *, *P* < 0.05; **, *P* < 0.01 (Mann-Whitney test comparing two groups).

The prime-boost using the malaria vaccine candidate TRAP (thrombospondin-related adhesion protein), expressed by adenoviral or modified vaccinia virus Ankara viral vectors (viral-vectored TRAP, or vvTRAP), combined with a regime consisting of adenovirus expressing CSP followed by a protein boost (Ad-P) CSP has previously been tested, showing enhancement in protection against a challenge with wild-type P. berghei compared to use of either antigen alone (8). Here, we applied a similar vaccination strategy using P. vivax antigens to assess protective efficacy against a challenge with the double-transgenic PvCSP-VK210/PvTRAP P. berghei described above; BALB/c mice were vaccinated as shown in Fig. 2A. High frequencies of *ex vivo* gamma interferon (IFN-γ) enzyme-linked immunosorbent spot (ELISpot) responses were induced against an immunodominant PvTRAP CD8^+^ epitope (Fig. 2B). Similarly, high titers of anti-CSP antibodies (Fig. 2C) were obtained, and no significant differences in either antibody titers or T-cell frequencies were observed between a single- and a double-component vaccine. No vaccine approach conferred sterile protection to any mouse following a parasite challenge (Fig. 2D). However, the combination of vvTRAP and Ad-P CSP, vvTRAP plus (vvCSP-PvCSP), significantly enhanced protective efficacy, measured as an increase in the time taken to reach 1% blood stage parasitemia compared to results with either component vaccine alone, confirming that, as with wild-type P. berghei, the combination of vivax vvTRAP and CSP antigens enhances protection against a sporozoite challenge even when equal magnitudes of adaptive humoral and cellular immune responses were induced, indicating the advantage of using two different vaccine candidates simultaneously for vaccination. To determine if higher levels of protection could be obtained, we next substituted vvCSP and PvCSP protein for Rv21, a P. vivax CSP preerythrocytic vaccine based on a hepatitis B virus-like particle (VLP), which has recently been shown to induce high levels of protective efficacy against a challenge with transgenic sporozoites expressing vivax CSP (15).

### Effect on immunogenicity of a combination of vvTRAP, Rv21, and two adjuvants.

**FIG 3:**
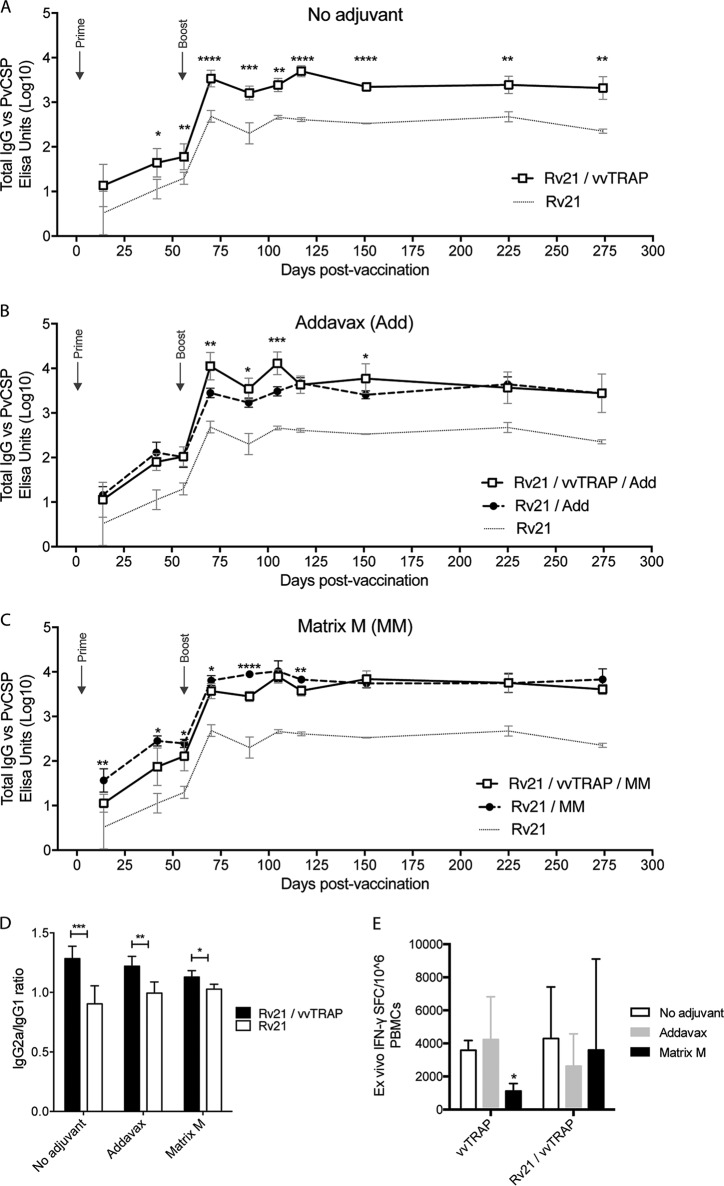
Adjuvanticity in a combination of Rv21 P. vivax CSP VLP with adenovirus and modified vaccinia Ankara viral vectors expressing P. vivax TRAP (vvTRAP). Inbred BALB/c mice (*n* = 6 per group) were vaccinated with combinations of CSP and vvTRAP as described in Table 1. Kinetics analysis of anti-CSP antibodies, measured by ELISA at the indicated time points, was determined with or without coadministration of vvTRAP. (A) Effect of the coadministration of Rv21 (PvCSP) with vvTRAP without addition of adjuvants. (B) Effect of AddaVax on anti-PvCSP antibodies induced by Rv21 alone or by Rv21+vvTRAP. (C) Effect of Matrix-M on anti-PvCSP antibodies induced by Rv21 alone or by Rv21+vvTRAP. (D) IgG2a/IgG1 ratios in BALB/c mice using Rv21 with or without vvTRAP and adjuvants. (E) Effect of Rv21, AddaVax, and Matrix-M on *ex vivo* IFN-γ responses against PvTRAP in mice. *t* tests were used for statistical analyses. *, *P* < 0.05; **, *P* < 0.01; ***, *P* < 0.001; ****, *P* < 0.0001.

We investigated various approaches to adjuvant immune responses to the P. vivax vaccine candidates CSP and TRAP. To this end, BALB/c mice were vaccinated with combinations of vvTRAP and Rv21 in the presence or absence of two adjuvants suitable for human use (Table 1). The combination consisted of a mixture of TRAP viral vectors (vvTRAP) delivered as an adenovirus expressing vivax TRAP with Rv21 presenting CSP used for priming and followed by a boost 8 weeks later using a combination of MVA-TRAP mixed with Rv21. Of interest, we observed a significant adjuvanting effect on the vivax CSP antibodies just by mixing vvTRAP with Rv21 at the doses indicated in Table 1 in the absence of any chemical adjuvant. This was evident both after a prime with adenovirus plus Rv21 and after a boost with MVA plus Rv21 and for a follow-up period of 275 days (Fig. 3A). Next, we studied the effect of adding two clinically relevant adjuvants to Rv21 or to the mixture of Rv21 plus vvTRAP (Rv21+vvTRAP). We first tested AddaVax, a squalene-based oil-in-water nanoemulsion with a formulation similar to that of MF59 (26). AddaVax-adjuvanted Rv21 increased significantly the anti-PvCSP titers after both a prime and homologous Rv21 prime/boost regimens compared to results with Rv21 alone. When AddaVax was added to the mixture of Rv21+vvTRAP, a significant increase in titers was seen after an MVA boost compared to results using Rv21 with AddaVax alone (Fig. 3B). This trend continued for up to 150 days, a time when responses became similar in both groups and remained comparable until the end of the study. Additionally, we tested Matrix-M, a saponin-based adjuvant (27, 28) that has been shown to enhance Rv21 immunity against a sporozoite challenge (15). As expected, Matrix-M significantly enhanced the anti-PvCSP responses elicited by Rv21 compared to those of the group with no adjuvant. However, the inclusion of viral vectors to the mixture resulted in a decrease in PvCSP-specific antibodies, which was evident at various time points after the prime or after the boost. The antigenic interference effect of viral vectors with Matrix-M appeared to be temporary as beyond 150 days postprime, anti-CSP titers in both groups receiving Matrix-M adjuvants were similar (Fig. 3C). Mixing vvTRAP with Rv21 significantly increased the IgG2a/IgG1 ratio, and the use of adjuvants also contributed to enhance this ratio in the absence of adjuvants (Fig. 3D). TRAP-specific CD8^+^ responses induced by viral vectors did not show major differences in the presence or absence of adjuvants, with the exception of a decrease in IFN-γ released by CD8^+^ T cells in the Matrix-M group, measured in an *ex vivo* IFN-γ ELISpot assay after a boost with a mixture (Fig. 3E). This confirms recent observations made in our laboratory (29).

**TABLE 1:** Vaccination regimes using viral vectors expressing TRAP (vvTRAP) and Rv21 in BALB/c mice

Vaccine regimen (adjuvant)^*a*^	Prime		Boost	
	Rv21-CSP (μg)	Ad-PvTRAP (IU)	Rv21-CSP (μg)	MVA-PvTRAP (PFU)
Rv21 + vvTRAP	1.5	1 × 10^8^	1.5	1 × 10^7^
Rv21	1.5		1.5	
vvTRAP		1 × 10^8^		1 × 10^7^
Rv21 + vvTRAP (AddaVax)	1.5	1 × 10^8^	1.5	1 × 10^7^
Rv21 (AddaVax)	1.5		1.5	
vvTRAP (AddaVax)		1 × 10^8^		1 × 10^7^
Rv21 + vvTRAP (Matrix-M)	1.5	1 × 10^8^	1.5	1 × 10^7^
Rv21 (Matrix-M)	1.5		1.5	
vvTRAP (Matrix-M)		1 × 10^8^		1 × 10^7^

a Adjuvant, where present, was used in both prime and boost. For all regimens, the prime-boost interval was 8 weeks.

### Efficacy of a preerythrocytic vivax vaccine is increased using a bivalent vaccine consisting of vvTRAP and PvCSP in VLPs.

**FIG 4:**
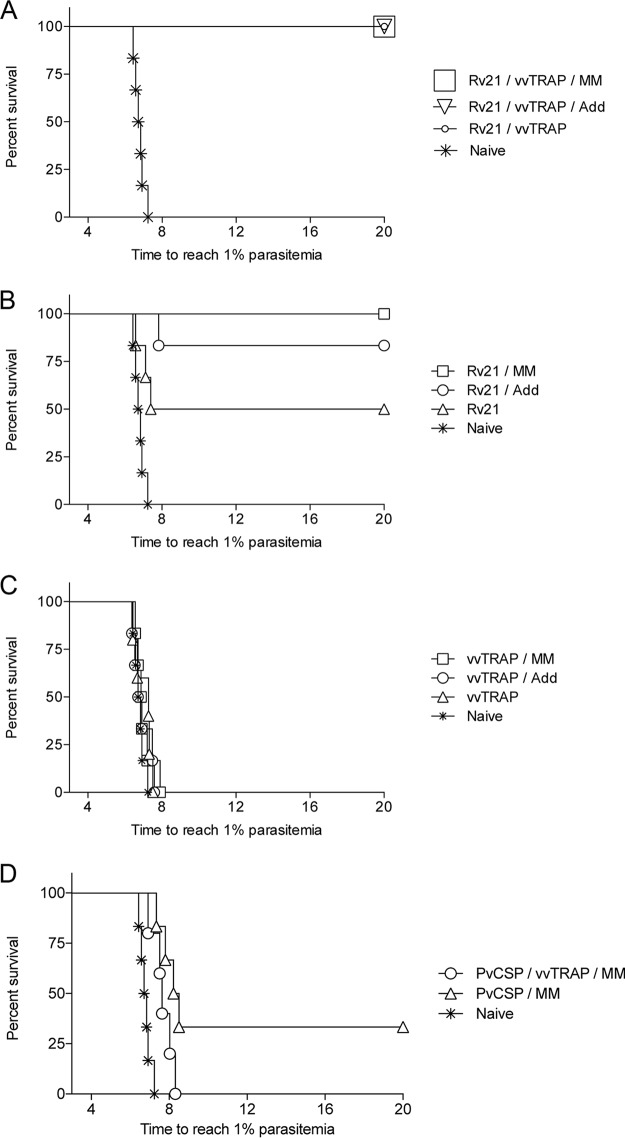
Protective efficacy against a transgenic sporozoite challenge using a combination of the Rv21 and vvTRAP, in the presence of Matrix-M or AddaVax adjuvants. Inbred BALB/c mice (*n* = 6 per group) vaccinated with Rv21 and/or vvTRAP as described in Table 1 were challenged by intravenous administration of transgenic P. berghei PvCSP-VK210/PvTRAP double-replacement parasites using a stringent challenge with 2,000 sporozoites. (A) Efficacy of a vaccine consisting of Rv21 combined with either vvTRAP or of vvTRAP plus the adjuvant Matrix-M (MM) or AddaVax (Add). (B) Efficacy of Rv21 in the presence of chemical adjuvants. (C) Efficacy of a prime-boost vaccination regimen using vvTRAP. (D) Efficacy of a P. vivax CSP protein in combination with vvTRAP and Matrix-M adjuvants.

The initial results indicating an adjuvant effect on P. vivax CSP by the use of a combination of viral vectors, virus-like particles (VLPs), Matrix-M, and AddaVax prompted us to investigate if the increased antibody titers resulted in enhanced protective efficacy against malaria infection. To this end, we challenged vaccinated mice with our newly developed chimeric PvCSP-VK210/PvTRAP sporozoites (Fig. 4). Unadjuvanted bivalent vaccines consisting of vvTRAP, mixed with only 1.5 μg of Rv21 VLP presenting PvCSP on its surface, protected 100% of the challenged mice, providing sterile efficacy with no signs of parasitemia for up to 20 days postchallenge. Inclusion of Matrix-M and AddaVax in the formulation retained the high protective levels of combined vaccines (Fig. 4A). Importantly, removing vvTRAP from the vaccine composition still yielded 100% sterile efficacy in Rv21 plus Matrix-M, while there was a trend toward reduction of efficacy in Rv21 plus AddaVax on a challenge (83.3% protection with AddaVax versus 100% with Matrix-M; *P* = 0.36) under conditions where only 50% of the mice were sterilely protected after vaccination with unadjuvanted Rv21 (Fig. 4B). In contrast, prime/boost vaccination with only vvTRAP did not protect mice against a sporozoite challenge, regardless of the presence or absence of adjuvants (Fig. 4C). Our results indicate a dose-sparing effect of the vaccines by administering Rv21 combined with either vvTRAP or adjuvants to enhance immunity against malaria without the need of increasing vaccine doses and open a possibility for the use of the combination as a fractional dose while retaining protection against malaria. Finally, we investigated if a protein in adjuvant had similar effects on protection and injected vivax CSP protein (pPvCS) in Matrix-M. Sterile protective efficacy was achieved in only 33% of the vaccinated mice (Fig. 4D) in contrast to 100% of protection elicited by Rv21 in Matrix-M, stressing the advantage of using a VLP over a soluble antigen. A summary of the survival efficacy for all groups is presented in Table 2.

**TABLE 2:** Summary of the survival efficacy induced by P. vivax CSP plus TRAP with vaccination regimens using vvTRAP, protein, or Rv21 in mice challenged by P. berghei sporozoites^*a*^

Vaccine regimen^*b*^	No. of protected mice/total no. of mice	Sterile protection (%)	Median survival (days)	Survival limit (days)	Mean survival ± SD (days)	SE
Naive	0/6	0	6.78	6.49–7.08	6.78 ± 0.28	0.12
vvTRAP	0/6	0	7.28	6.46–7.62	7.12 ± 2.5	0.21
Rv21	3/6	50	13.69	6.06–20.98	13.52 ± 7.1	2.9
Rv21+vvTRAP	6/6	100	20	20.0–20.0	20.0 ± 0	0
vvTRAP+MM	0/6	0	6.89	6.52–7.53	7.03 ± 0.48	0.20
Rv21+MM	6/6	100	20	20.0–20.0	20.0 ± 0	0
Rv21+vvTRAP+MM	6/6	100	20	20.0–20.0	20.0 ± 0	0
pCS+MM	3/6	33	8.36	5.44–18.51	11.98 ± 6.23	2.54
vvTRAP+pCS+MM	0/6	0	7.64	7.02–8.35	7.68 ± 0.53	0.24
vvTRAP+Add	0/6	0	6.81	6.41–7.46	6.9 ± 0.5	0.20
Rv21+Add	5/6	83.3	20	12.7–23.2	20.0 ± 0	2.03
Rv21+vvTRAP+Add	6/6	100	20	20.0–20.0	20.0 ± 0	0

a Vaccinated BALB/c mice were challenged with 2,000 sporozoites of the PvCSP-PvTRAP 2207 cl1 strain, as described in the legend of Fig. 4.

b Adjuvants used were Matrix-M (MM) and AddaVax (Add).

### A combination of Rv21 VLP and viral vectors requires vivax antigens in both platforms to enhance protective efficacy against a sporozoite challenge.

**FIG 5:**
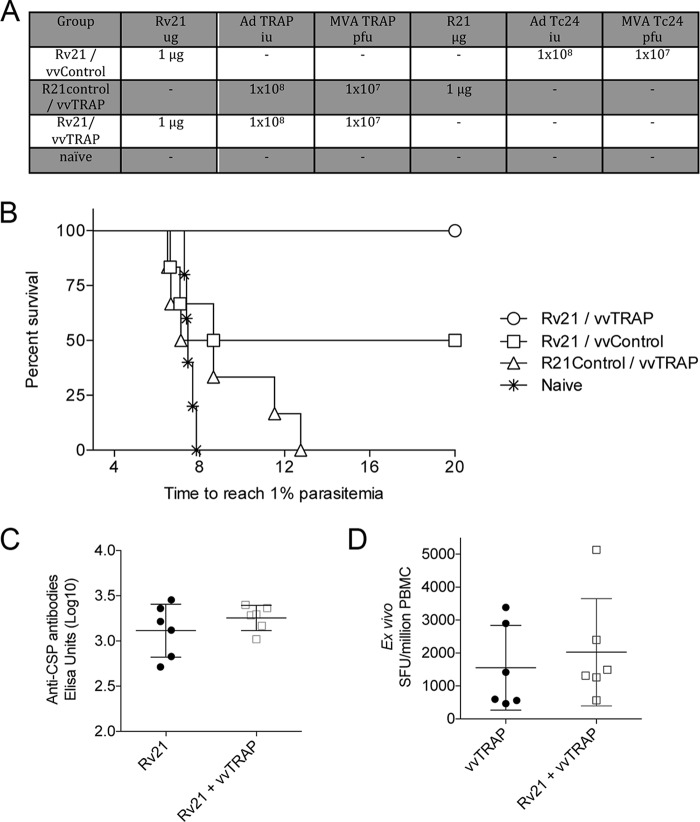
Assessment of vaccine efficacy using a combination of Rv21 with viral-vectored vaccines expressing P. vivax TRAP (vvTRAP) or an unrelated transgene (vvControl). (A) Table indicating the vaccination components in an 8-week prime-boost regimen, utilized to compare the efficacy against a sporozoite challenge with a combination of a P. vivax VLP (Rv21), a P. falciparum VLP (R21) with vvTRAP, or an unrelated nonmalaria antigen vvControl. (B) Survival of mice (*n* = 6) after a sporozoite challenge with the vaccination regimens described in panel A. (C) Titer of anti-PvCSP antibodies by Rv21 or a combination of Rv21 plus vvTRAP. (D) *Ex vivo* IFN-γ responses against TRAP measured by ELISpot assay. SFU, spot-forming units.

In the experiments described in the sections above, anti-PvCSP antibody responses induced by injection of 1.5 μg of Rv21 were adjuvanted by coimmunization with vvTRAP. The adjuvanting effect, unless properly controlled for, would make the interpretation of enhancement of protection from Rv21 and vvTRAP coadministration difficult as it would not be clear if any enhancement in protection derived from the increased breadth of response to multiple antigens or merely from an improved immunogenicity to PvCSP titers resulting from inclusion of vvTRAP in the vaccine formulation. To this end, lower doses of 1 μg of Rv21 were mixed with viral vectors expressing an unrelated Trypanosoma cruzi antigen, Tc24 (30), while a control group received vvTRAP mixed with R21 presenting P. falciparum CSP (Fig. 5A) (31). This resulted, as desired, in identical anti-CSP titers in the Rv21 and Rv21+vvTRAP groups and identical TRAP-specific ELISpot assay responses in the vvTRAP and Rv21+vvTRAP groups, thus eliminating the possibility that an enhancement of protection in the Rv21+vvTRAP group compared to protection of the single-component groups was due to vvTRAP enhancing antibody responses to PvCSP (Fig. 5C and D). Complete protection was achieved only in the group in which Rv21 and vvTRAP were combined (Fig. 5B). In contrast, when Rv21 was mixed with viral vectors expressing an unrelated Chagas Tc24 antigen (vvControl), only 50% of the mice were completely protected, and no sterile protection was afforded upon vaccination with vvTRAP mixed with R21 presenting P. falciparum CSP.

## DISCUSSION

Here, it is demonstrated that immunity against malaria using parasites expressing P. vivax antigens is enhanced by a combination of two preerythrocytic vaccine candidates, PvCSP and PvTRAP. This is achieved in mice using a newly developed double-transgenic P. berghei parasite in which the endogenous PbCSP and PbTRAP were replaced by their P. vivax homologues. Our initial observations using a combination of a P. vivax CSP protein with vvTRAP resulted in suboptimal protection against a sporozoite challenge with the double-transgenic parasite. Importantly, when the CSP protein was replaced with the virus-like particle (VLP) Rv21, 100% protection was obtained even in the absence of any adjuvant. Our results open an opportunity to replace an adjuvant by viral vectors expressing other malaria antigens to enhance protective immunity through the improvement of immune responses or by targeting additional malaria antigens. This is important due to the limited number of adjuvants that are currently available for human use (32). Greater availability of immunostimulatory alternatives could contribute to reducing reactogenicity as this is the inevitable price for improving immunogenicity in adjuvanted vaccines (33). Moreover, our results support the use of fractional doses of a VLP due to a dose-sparing effect when Rv21 and viral vectors were combined, and while an adjuvant affects the cost of a vaccine, replacing it with a viral vector may not significantly alter the total cost of a vaccination approach and gives an opportunity to assess alternative methods to limit reactogenicity while improving the breadth of responses, thus enhancing immunity against malaria.

Our results support the notion that protective immunity against malaria may require harnessing humoral and cellular responses against multiple antigens (7), which has been shown in earlier studies where a combination the two preerythrocytic antigens, CSP and TRAP, has enhanced protection against mouse malaria in a P. berghei mouse sporozoite challenge (8). Such studies stressed the importance of the protective effect of monoclonal antibodies against CSP and how, at suboptimal doses, protection was rescued by viral vector vaccination against P. berghei TRAP. In the current study, we have confirmed this observation using the P. vivax proteins as vaccine targets by maximizing anti-CSP antibody responses through the use of the Rv21 VLP and T-cell responses using viral vectors expressing TRAP. Similar observations have been made for P. falciparum. In a recent phase IIa clinical trial (9), a leading P. falciparum malaria vaccine was assessed for protective efficacy against a sporozoite challenge. This consisted of a CSP-based VLP RTS,S in combination with viral-vectored ME-TRAP. In that study, the combination showed evidence of being more efficacious (82.4%) than RTS,S alone (75%). This demonstrates a difficulty also encountered in the design of the present study: establishing that the combination of TRAP and CSP enhances efficacy requires that both regimens alone elicit suboptimal protection. A mathematical model (34) predicts that combining an RTS,S vaccine of 50% efficacy with a viral-vectored ME-TRAP regime with 21% efficacy would confer protective efficacy of 97%. The murine evidence presented here and elsewhere (16, 23) is consistent with this prediction and suggests that a strategy of combining subunit vaccines could prove crucial in developing a malaria vaccine of the desired 75% efficacy (35) and yet provide a dose-sparing effect where improved efficacy with low doses of vaccines could contribute to a decrease in vaccine costs.

Our results also indicate that an improvement in protective efficacy requires the combination of Rv21 with vivax vvTRAP to act synergistically in order to completely eliminate infection since the use of viral vectors expressing unrelated transgenes does not produce improved immunity against malaria. This indicates that augmenting the breadth of responses to multiple antigens plays an important role in the increase in protective efficacy seen here with Rv21 and vvTRAP coadministration. This supports the observations made previously stressing the major challenge posed by the complexity of the malaria parasite that transitions from extracellular to intracellular stages during the life cycle in the mammalian host, thus requiring harnessing cellular and humoral responses against more than one antigen to induce protective immunity (7).

It is possible that a CSP/TRAP combination regime without adjuvants could be used in future P. vivax clinical trials, given that viral vectors have been shown here to possess as potent an adjuvant effect as the other adjuvants tested. It remains to be seen whether the adjuvanting effects of VLPs and viral vectors on each other in the absence of adjuvant are recapitulated in phase I and II trials.

When considering the implications of these results on the choice of adjuvant for a CSP/TRAP combination vaccine, we must also consider the long-term effects of adjuvants on immune responses. Antigenic interference was not seen in the medium to long term with Matrix-M or AddaVax in this study. Indeed, 150 days after priming, anti-CSP titers were highest in Matrix-M-vaccinated groups. Given that the malaria research community is aiming for a vaccine which remains 75% effective for 2 years (35), longevity of protection may prove more important than short-term antigenic effects on interference in the decision about the most appropriate adjuvant to use. In this regard, Matrix-M and AddaVax are equally good in the induction of protective immunity against malaria, at least in terms of antibody titers.

In summary, the combination of two P. vivax antigens, CSP and TRAP, using appropriate platforms for each candidate (a VLP for CSP and viral vectors for TRAP), improves protective efficacy compared to use of either alone. Improved immunogenicity and protective efficacy was also seen with the use of AddaVax and Matrix-M adjuvants in combination with Rv21. This study demonstrates the potential of combining subunit antigens using suitable platforms as a method of enhancing the protective efficacy of malaria vaccines.

## MATERIALS AND METHODS

### Generation of DNA constructs and genotyping of the double-chimeric parasite line PvCSP/PvTRAP.

Two reference WT lines of P. berghei ANKA were used to construct a double-chimeric parasite: the wild-type (WT) reference line cl15cy1 of P. berghei ANKA (36) and the reporter PbANKA parasite line PbGFP-Luc_con_ (676m1cl1). The PbGFP-Luc_con_ parasite expresses a fusion protein of GFP (mutant 3) and firefly luciferase (LUC-IAV) under the constitutive *eef1a* promoter and is selectable marker (SM) free (37). The reporter cassette is integrated into the neutral *230p* locus (Eukaryotic Pathogen Database [EuPathDB] accession number PBANKA_030600). For details of PbGFP-Luc_con_, see the RMgm (Rodent Malaria genetically modified) database (entry RMgm-29 [http://www.pberghei.eu/index.php?rmgm=29]).

For the generation and genotyping of the chimeric parasites, we used Swiss mice (OF1/ico, construct 242; 6 weeks old; 25 to 26 g) (Charles River). All animal experiments performed at the Leiden University Medical Center (LUMC) were approved by the Animal Experiments Committee of the Leiden University Medical Center (DEC 12042). The Dutch Experiments on Animals Act was established under European guidelines (European Union directive no. 86/609/EEC regarding the Protection of Animals used for Experimental and Other Scientific Purposes).

The double-chimeric line PvCSP-VK210/PvTRAP in which both the *csp* (EuPathDB accession number PBANKA_040320) and the *trap* (EuPathDB accession number PBANKA_134980) CDSs of P. berghei were replaced with *csp* (EuPathDB accession number PVX_119355) and *trap* (NCBI accession number XM_001614097) genes of P. vivax was generated by replacing the *Pbtrap* gene with *Pvtrap* in a single chimeric line in which the *Pbcsp* gene had been previously replaced by the VK210 allele of *Pvcsp*. The single-chimeric line, PbANKA-PvCSP-VK210(r)_PbCSP_ (line 2196cl1), had been generated using the gene insertion/marker out (GIMO)-based transfection technology as described previously (15) and is free of a drug-selectable marker. In addition, it contains the GFP-luciferase fusion gene as a reporter. The absence of a drug-selectable marker allowed the replacement of the *Pbtrap* gene with the *Pvtrap* gene in parasites of the PbANKA-PvCSP-VK210(r)_PbCSP_ line in a single transfection experiment using the *trap* replacement construct described by Bauza et al. (16). This construct contains a synthetic *trap* allele composed of the protein coding sequence of P. vivax TRAP (NCBI accession number XM_001614097) from the Salvador I strain, which was codon optimized for expression in P. berghei using GeneOptimizer software. In addition, this construct contains a positive/negative selectable marker (SM) cassette with the human *dhfr* and yeast *fcu* (h*dhfr*::y*fcu*) fusion gene. Transfection of PbANKA-PvCSP-VK210(r)_PbCSP_ parasites with the *Pvtrap* replacement construct and positive selection with pyrimethamine were performed using standard methods for transfection of P. berghei (36). This resulted in selection of double-chimeric parasites (line 2207). Selected double-chimeric parasites were cloned by the method of limiting dilution (38), resulting in the PbANKA-PvCSP-VK210(r)_PbCSP_+PvTRAP(r)_PbTRAP_+SM line (2207 cl3). Correct integration of the constructs into the genome of the double-replacement gene [DRG] chimeric parasite was analyzed by diagnostic PCR analysis on gDNA and Southern analysis of pulsed-field gel electrophoresis (PFGE)-separated chromosomes as described previously (36). Primers used for PCR genotyping are listed in Table S1 in the supplemental material.

Subsequently, we recycled the positive-negative SM cassette from the parasites of line 2207 cl3 by applying negative selection by providing 5-fluorocytosine (5-FC) in the drinking water of mice (39). Negative selection and removal of the h*dhfr*::y*fcu* SM cassette resulted in selection of PbANKA-PvCSP-VK210(r)_PbCSP_+PvTRAP(r)_PbTRAP_ parasites (2207 cl3m0). Selected parasites were cloned by the method of limiting dilution (38) resulting in parasite line 2207 cl3m0cl1. This double-chimeric line PbANKA-PvCSP-VK210(r)_PbCSP_+PvTRAP(r)_PbTRAP_ is referred to as PvCSP-VK210/PvTRAP parasites.

Correct integration of the constructs into the genome of double-chimeric parasites was analyzed by diagnostic PCR analysis on gDNA and Southern analysis of pulsed-field gel electrophoresis (PFGE)-separated chromosomes as described previously (37). Primers used for PCR genotyping are listed in Table S1.

### Phenotyping of the double-chimeric parasites.

Growth of blood stages of the reporter and chimeric P. berghei parasites was determined during the cloning period, as described previously (36, 40). Feeding of A. stephensi mosquitoes, determination of oocyst production, and sporozoite collection were performed as described previously (40). Expression of PvCSP-VK210 and PvTRAP antigens in sporozoites of the chimeric parasites was analyzed by immunofluorescence staining assay (IFA), using two anti-P. vivax antigen monoclonal antibodies, anti-PvCSP-VK210 (obtained through BEI Resources, NIAID, NIH; hybridoma 2E10.E9 anti-Plasmodium vivax circumsporozoite protein, catalogue no. MRA-185, contributed by Elizabeth Nardin) (diluted 200 times) or anti-PvTRAP polyclonal antibodies elicited by viral vector vaccination. In addition we used anti-PbCSP 3D11 (41) antibodies as a control (diluted 1,000 times) and serum from mice vaccinated with ChAd63-MVA expressing PbTRAP (diluted 50 times). Purified sporozoites were fixed with 4% paraformaldehyde in phosphate-buffered saline (PBS) for 20 min on ice, washed three times with PBS, and blocked with 20 μl of 10% fetal calf serum (FCS) plus 1% bovine serum albumin (BSA) in PBS for 30 min at room temperature. The excess blocking medium was removed, followed by the addition of 20 to 25 μl of primary monoclonal antibody in 10% FCS–1% BSA in PBS (blocking medium) for 1 to 2 h at room temperature or overnight at 4°C. After incubation, the primary antibody was removed, and the slides were washed three times with PBS, followed by staining with a secondary antibody, Alexa Fluor 488 goat anti-mouse IgG (catalog number A-11001; Life Technologies), diluted 800 times in 10% FCS–1% BSA in PBS (blocking medium) for 1 h at room temperature. After three washes with PBS, nuclei were stained with 2% Hoechst-33342 (4082S; Cell Signaling Technology) in PBS for 10 min at room temperature, washed twice with PBS, and left to air dry; this was followed by the addition of fluorescence mounting medium (code S3023; Dako) before complete dry out. Coverslips were mounted onto the slides, and the slides were sealed with nail polish and left to dry overnight in the dark. The parasites in both blue and green channels were analyzed using a DMI-300B Leica fluorescence microscope, and images were processed using ImageJ software.

### Viral vector vaccines.

Mice were primed with simian adenoviral vector 63 (ChAd63) encoding PvTRAP, as described earlier (16), and PvCSP VK210 (15) and 8 weeks later boosted with modified vaccinia virus strain Ankara (MVA) carrying the same transgene at a dose of 1 × 10^7^ PFU (15, 16). A control viral vector was designed and constructed to express Tc24-IMX (a Trypanosoma cruzi antigen; GenBank accession number U70035.1). Briefly, the Tc24 gene from Trypanosoma cruzi was synthesized by GeneArt (Germany) and cloned into the plasmid pMono2 using the restriction sites Acc651 and BamHI. Tc24 was cloned into ChAd63, and the presence of the Tc24 gene was confirmed by PCR, while the integrity of the antigenic DNA sequence and absence of contaminating adenovirus were confirmed by PCR followed by restriction fragment length polymorphism (RFLP).

The virus was titrated to obtain the number of infectious units (IU) per milliliter and assayed by spectrophotometry to quantify the number of virus particles per milliliter. The sterility of the virus was also confirmed by inoculation of tryptic soy broth (TSB) with 10 μl of purified virus and incubation for 3 days at 35°C. Quality control tests indicated infectious units by titration of 1.0E+11 IU/ml, a virus particle (VP) concentration of 7.2E+12 VP/ml, and a particle/infectious units (P/I) ratio of 70.3 at a dose of 1 × 10^8^ IU. To construct MVA-Tc24, the gene was cloned into a plasmid (p2773) suitable for MVA recombination. The virus and plasmid were recombined, and the cell lysate was harvested and used at 1:10 and 1:50 dilutions to infect chicken embryonic fibroblasts (CEF). CEF cells were MoFlo sorted into 96-well plates. Recombinant virus was purified by selecting individual plaques to be subsequently cultured to eliminate parental virus, and purity was determined through amplification of the DNA sequence by PCR followed by RFLP. The virus was then amplified to confirm the presence of the antigen in question and lack of original, parental virus expressing the red fluorescent protein in the final stock. Sterility of the virus was confirmed by inoculation of TSB with 10 μl of purified virus and incubation for 3 days at 35°C. The numbers of PFU were calculated upon titration, yielding 4.6 × 10^8^ PFU/ml. Additional control viral vectors expressing ovalbumin (Ova) and GFP have been published previously, such as AdHu5 Ova (42), ChAd63 Ova, and MVA GFP (8).

### Rv21 virus-like particle vaccine.

Rv21 is a chimeric fusion of the central repeat regions of PvCSP-VK210 (NCBI accession no: P08677) and VK247 (NCBI accession no: M69059) and the C terminus of PvCSP-VK210, fused to the N terminus of hepatitis B surface antigen (HepBsAg) and expressed in Pichia pastoris (15). A gene containing a chimeric CSP sequence comprising the repeat regions VK210 and VK247, followed by the C-terminal sequence (210/247C), was designed to be fused to HepBsAg. This VLP was described previously (15). Characterization of the Rv21 VLP was made by Western blotting using anti-PvCSP-VK210 (obtained through BEI Resources, NIAID, NIH; hybridoma 2E10.E9 anti-Plasmodium vivax circumsporozoite protein, catalogue mo. MRA-185, contributed by Elizabeth Nardin) and anti-PvCSP-VK247 (obtained through BEI Resources, NIAID, NIH; hybridoma 2F2 anti-Plasmodium vivax circumsporozoite protein, catalogue no. MRA-184, contributed by Elizabeth Nardin) primary mouse antibodies, diluted in 3% BSA-PBS to 1:200, 1:20,000, and 1:20,000. A secondary donkey anti-mouse alkaline phosphatase (AP) conjugate in 3% BSA-PBS and Sigmafast BCIP/NBT (5-bromo-4-chloro-3-indolylphosphate/nitroblue tetrazolium) tablets (Sigma-Aldrich) were used for development.

### Vaccination.

All viral vector, VLP, and protein vaccines were administered intramuscularly in endotoxin-free PBS. Combinations of vaccines were mixed prior to administration (called coadministration in this paper) unless otherwise stated.

### Animals.

Female inbred BALB/c (H-2^d^) mice were used for the assessment of immunogenicity and protection after challenge. Six to 8 mice per group were used for the studies. Tuck-ordinary (TO) outbred mice were used for parasite production and transmission. The mice were purchased from Harlan (United Kingdom).

### Ethics statement.

All animals and procedures were used in accordance with the terms of the United Kingdom Home Office Animals Act Project License. The procedures were approved by the University of Oxford Animal Care and Ethical Review Committee (PPL 30/2414).

### Protein production.

PvTRAP protein used for enzyme-linked immunosorbent assays (ELISAs) was prepared using HEK293 cells as described earlier (16, 43). PvCSP was produced as described earlier (15). Use of Ova protein was described earlier (44).

### *Ex vivo* IFN-γ ELISpot assay.

*Ex vivo* IFN-γ ELISpot assays were carried out using peripheral blood mononuclear cells (PBMCs) isolated from blood as previously described (8, 16). Twenty-amino-acid peptides from PvTRAP previously shown to be immunodominant in BALB/c mice (ITKVIPMLNGLINSLSLSRD) (16) (ThinkPeptides) or peptide pools of 20 amino acids representing the full-length protein and overlapping by 10 amino acids (Mimotopes) were used to stimulate PBMCs at a final concentration of 5 μg/ml. MAIP ELISpot plates (Millipore) were used to plate cells. Anti-mouse IFN-γ monoclonal antibody and development reagents were used according to the manufacturer's specifications (Mabtech).

### Whole IgG ELISA, avidity ELISA, and IgG subclass ELISA.

Enzyme-linked immunosorbent assays (ELISAs) measuring total IgG were carried out as described previously (45), and serum antibody endpoint titers were taken as the *x* axis intercept of the dilution curve at an absorbance value 3 standard deviations greater than the optical density at 405 nm (OD_405_) for serum from a naive mouse.

For standard-curve ELISAs, plates were prepared as for endpoint ELISAs, and then serum was diluted between 1:500 and 1:20,000 in PBS-Tween before application in triplicate at 50 μl/well. A standard curve was produced in duplicate on each plate by 3-fold serial dilution from 1:750 (for PvCSP) or 2-fold serial dilution from 1:100 (for PvTRAP) through 10 wells using stock sera from Rv21 (15) and Ad-M PvTRAP-vaccinated mice (16). Following 2 h of incubation at room temperature, plates were treated as for endpoint ELISAs, with plates scanned at 405 nm after 14 min for PvCSP-coated plates and 18 min for PvTRAP-coated plates. MARS (Multivariate Adaptive Regression Splines) software was used to obtain four-parameter logistic values of the standard curve on each plate for analysis and expression of serum OD values in terms of arbitrary ELISA units (EU) relative to the standard curve.

For avidity ELISAs, standard-curve ELISAs were performed as described above except that after 2 h of incubation of the serum at room temperature, plates were washed six times in PBS-Tween, and 100 μl of 7 M urea was added to serum wells for 10 min. Plates were then washed six times in PBS-Tween, and the standard-curve ELISA was carried out as before. The avidity index was calculated as the percentage of log_10_ EU of urea-treated compared to untreated serum samples, following the procedure described in (46).

For IgG subclass ELISAs, standard-curve ELISAs were performed at the end of the study, day 275, using a Bio-Rad Mouse Typer Sub-Isotyping kit according to the manufacturer's protocol (Bio-Rad) with the modification that after serum incubation, subclass-specific rabbit anti-mouse IgG was applied at 50 μl per well in triplicate. Serum for a standard curve was incubated with kappa-chain-specific IgG. After 1 h of incubation, plates were washed six times in PBS-Tween, and goat anti-rabbit horseradish peroxidase conjugate was applied (50 μl/well; 1:3,000). After 1 h plates were washed six times in PBS-Tween and developed for 14 min (PvCSP-coated plates) or 18 min (PvTRAP-coated plates) using 100 μl/well peroxidase substrate solution (Bio-Rad). Reactions were stopped by the addition of 100 μl/well of 2% oxalic acid.

### Parasite production and challenge.

Wild-type and transgenic parasites used to challenge mice were produced at the Jenner Institute insectary. Female Anopheles stephensi mosquitoes were fed on infected TO mice. Exflagellation was first confirmed, and mosquitoes were exposed to anesthetized infected mice for 15 min. The mosquitoes were then maintained for 21 days in a humidified incubator at a temperature of 19 to 21°C on a 12-h night-day cycle and fed with a fructose–*p*-aminobenzoic acid (PABA) solution. At 21 days salivary glands were dissected from mosquitoes into Schneider's medium (Pan Biotech), and sporozoites were gently liberated using a glass homogenizer. Sporozoites were diluted to the required concentration for 100 μl of intravenous injection into the tail vein of the mouse.

### Adjuvants.

When used, adjuvants were mixed and coinjected with vaccines. AddaVax was used at 25 μl per dose; Matrix-M was used at 5 μg per dose. AddaVax (InvivoGen) was kindly provided by the Jenner Institute adjuvant bank by Anita Milicic, and Matrix-M was obtained from Novavax AB.

### Statistical model for parasitemia prediction.

To extrapolate the liver-to-blood parasite load or predict the time to 1% blood stage infection, a linear regression model was used as described previously (47). Briefly, blood parasite counts were obtained for 3 to 5 consecutive days starting on day 4 after challenge. Blood smears were stained with Giemsa stain, and percentages of parasitemia were calculated in all animals. The log_10_ value of the calculated percentage of parasitemia was plotted against time after challenge, and Prism, version 6 (GraphPad Software), for Mac OS X was used for generating a linear regression model on the linear part of the blood stage growth curve.

### Statistical analysis.

For all statistical analyses, GraphPad Prism, version 6.0, for Mac OS X was used unless otherwise indicated. Prior to statistical analysis to compare two or more populations, the Kolmogorov-Smirnov test for normality was used to determine whether the values followed a Gaussian distribution. An unpaired *t* test was employed to compare two normally distributed groups, whereas a Mann-Whitney rank test was used to compare two nonparametric groups. If more than two groups were present, nonparametric data were compared using a Kruskal-Wallis test with Dunn's multiple-comparison posttest, whereas normally distributed data were analyzed by one-way analysis of variance (ANOVA) with Tukey's multiple-comparison posttest. Correlation strength was assessed using either Pearson's or Spearman's test, as indicated in Results. Kaplan-Meier survival curves were used to represent protective efficacy against challenge with P. berghei parasite lines. All ELISA titers were log_10_ transformed before analysis.

## Supplementary Material

Supplemental file 1

Supplemental file 2
