# Supplementary material for: Tailoring a Plasmodium vivax Vaccine To Enhance Efficacy through a Combination of a CSP Virus-Like Particle and TRAP Viral Vectors
Source: Infect Immun. 2018 Aug 22;86(9):e00114-18. doi: 10.1128/IAI.00114-18 (PMC6105880; doi:10.1128/IAI.00114-18)
Supplement: Supplemental file 1 [file zii999092538s1.pdf]

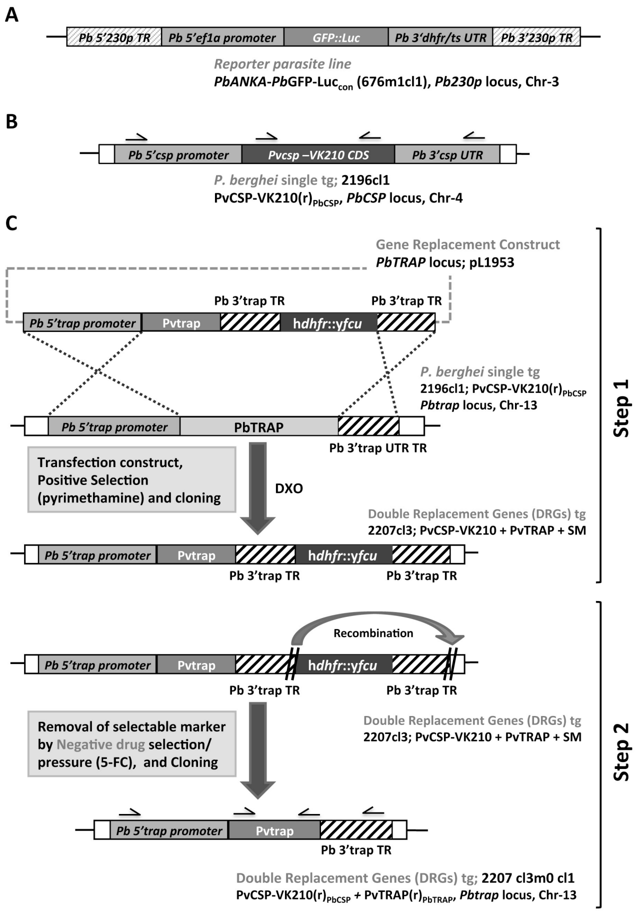

Figure S1: Generation of the double transgenic parasite PvCSP+PvTRAP, line 2207 cl3m0 cl1.

(A) Schematic representation of the transgenic Pb230p locus of the reporter PbANKA parasite line PbGFP-Luceef1α (676m1cl1) used to generate the transgenic parasite line PvCSP-VK210(r)$_{PbCSP}$ (2196 cl1). (B) Schematic representation of the PvCSP-VK210(r)$_{PbCSP}$ (2196 cl1) expressing the *P. vivax* CSP-VK210 coding sequence (CDS) under the control of the endogenous *PbCSP* regulatory sequences in chromosome 4. (C) Schematic representation of the generation of the transgenic parasite line PvCSP+PvTRAP (2207 cl3m0 cl1), upon cloning of *PbTRAP* into chromosome 13 in PvCSP-VK210(r)$_{PbCSP}$ 2196 cl1. *1st step*: Construct 1; pL1953 was used to replace the *PbTRAP* CDS with PvTRAP, resulting in the generation of line 2207cl3; *Pb*ANKA-PvCSP-VK210+PvTRAP+Selectable Marker. *2nd step:*

Removal of the selectable marker by negative drug selection using 5-Fluorocytosine (5-FC), yielding the double transgenic parasite *Pb*ANKA-PvCSP+PvTRAP (2207 cl3m0 cl1). This line contains both *P. vivax* CSP-VK210 (not shown) and TRAP expressed under the control of their equivalent endogenous regulatory sequences PbCSP and PbTRAP. Black arrows: location of primers used for diagnostic PCR.

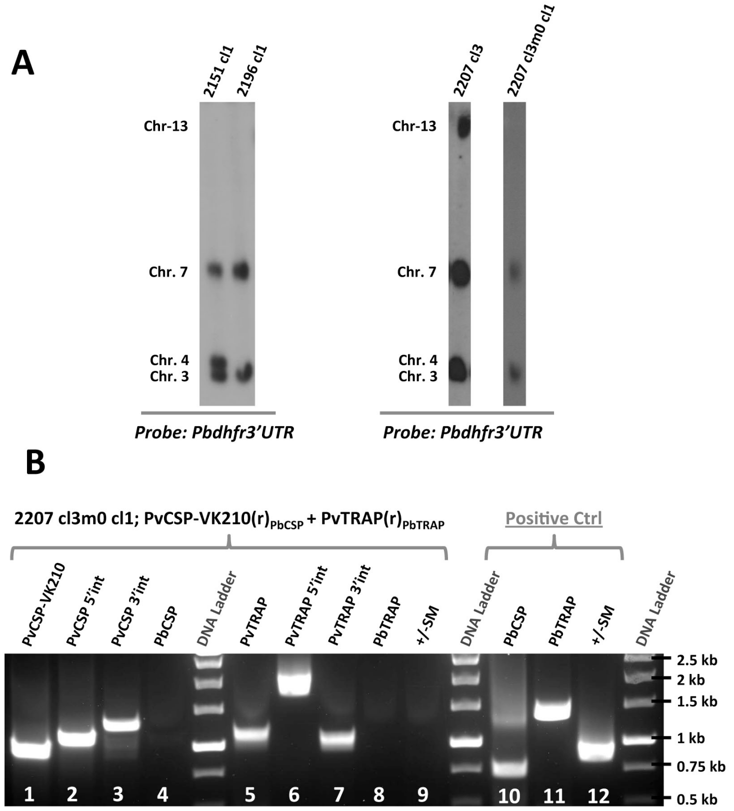

Lane	*PCR product*	*Primers*		*Size (bp)*
		F	*R*	
1	PvCSP-VK210	*1229*	*1230*	*1,020*
2	PvCSP 5'int	*1046*	*1230*	*1,161*
3	PvCSP 3'int	*1229*	*1047*	*1,383*
4	PbCSP	*1054*	*1055*	*NA*
5	PvTRAP	*1233*	*1234*	*1,219*
6	PvTRAP 5'int	*1114*	*1235*	*1,983*
7	PvTRAP 3'int	*1236*	*1117*	*1,154*
8	PbTRAP	*1118*	*1119*	*NA*
9	+/-SM	*1048*	*1049*	*NA*
10	PbCSP *Ctrl*	*1054*	*1055*	*774*
11	PbTRAP *Ctrl*	*1118*	*1119*	*1,532*
12	+/-SM *Ctrl*	*1048*	*1049*	*927*

Figure S2. Genotype analysis of the transgenic parasite *Pb*ANKA-PvCSP+PvTRAP, line 2207 cl3m0 cl1 and intermediate lines.

(A) Southern blot analysis*. Left panel*: Hybridisation of chromosomes of the two background lines 2151 cl1 (*Pb*ANKA-*PbCSP GIMO*) and 2196 cl1 (*PbANKA*-PvCSP-VK210(r)$_{PbCSP}$) with the 3′UTR *Pbdhfr/ts* probe. For *2151 cl1*, correct integration of the *SM* in the right locus in Chr-4 was confirmed by using the 3′UTR *Pbdhfr/ts* probe, which hybridizes in Chr-3, Chr-4 and Chr-7. Correct integration of the *PvCSP-VK210* expression construct into the GIMO locus was confirmed by the absence of the

h*dhfr::*y*fcu* SM cassette from chr.-4 in the cloned transgenic parasite line (2196 cl1). *Right panel*: Hybridisation of chromosomes of the two *Pb*ANKA-PvCSP+PvTRAP lines with and without the h*dhfr::*y*fcu* SM cassette, *2207cl3* and *2207 cl3m0 cl1*, respectively with the 3′UTR *Pbdhfr/ts* probe. Correct integration of the *PvTRAP* expression construct was confirmed by showing positive hybridization in chr. 13 of the *2207 cl3*. Correct recombination and removal of the h*dhfr::*y*fcu* SM cassette was confirmed by negative hybridization of 3′UTR *Pbdhfr/ts* probe in chr. 13, while this probe hybridizes to the GFP-Luc reporter-cassette in chr. 3 (Figure S1,A) and to the endogenous *Pbdhfr/ts* on chr. 7. (B) Genotype analysis by diagnostic PCR analysis of the transgenic parasite line *2207 cl3m0 cl1* to confirm the correct integration of both *PvCSP-VK210* and *PvTRAP* expression cassettes. Correct integration is shown by the absence of the h*dhfr*::y*fcu* SM, the presence of the *PvCSP-VK210* CDS and *PvTRAP* CDS.
