# Supplementary material for: Tailoring a Plasmodium vivax Vaccine To Enhance Efficacy through a Combination of a CSP Virus-Like Particle and TRAP Viral Vectors
Source: Infect Immun. 2018 Aug 22;86(9):e00114-18. doi: 10.1128/IAI.00114-18 (PMC6105880; doi:10.1128/IAI.00114-18)
Supplement: Supplemental file 2 [file zii999092538s2.pdf]

Table S1: Primers for genotyping the PvCSP+PvTRAP double transgenic parasite

Primer No.	Description	Primer sequences
1046	Pb5'CSP pro Int. F	TTGATAACCCTCACATAAGACAATCC
1047	Pb3'CSP UTR Int. R	TCGATATCGTCATAGCAAGTTAACTAC
1048	hDHFR-yFCU (+/-SM) F	ATCATGCAAGACTTTGAAAGTGAC
1049	hDHFR-yFCU (+/-SM) R	CATCGATTCACCAGCTCTGAC
1054	PbCSP F	CCAAAGGAACTTAAACGAGCTATG
1055	PbCSP R	CTTATACCAGAACCACATGTTACG
1114	Pb5'TRAP pro Int. F	AAATTGCCCCCTTTTTTGTGTTC
1117	Pb3'TRAP UTR Int. R	AATGACTCCAGACATAATAACACAGATATG
1118	PbTRAP F	ATGGCTCAGGAAGTATTGGTC
1119	PbTRAP R	ACCTATGCATCCAATTATAGCTAATC
1229	PvCSP-VK210 F	TTATTTCCAACACATTGTGGACATAATG
1230	PvCSP-VK210 R	AGCACATTTATCCATTGTACATACATCTG
1233	PvTRAP F	ACAAATATGACAGCAGCATTAGATGAAG
1234	PvTRAP R	CTTCATCAAATGGTGCTGGTTC
1235	PvTRAP 5'int. R	TCTAATGCTGCTGTCATATTTGTTGTTC
1236	PvTRAP 3'int. F	ATAGTGCAAGAGATAGATATGCAAGAC
